# Plasma carnitine concentrations in Medium‐chain acyl‐CoA dehydrogenase deficiency: lessons from an observational cohort study

**DOI:** 10.1002/jimd.12537

**Published:** 2022-07-17

**Authors:** Emmalie A. Jager, Merit Schaafsma, Melanie. M. van der Klauw, M. Rebecca Heiner‐Fokkema, Terry G. J. Derks

**Affiliations:** ^1^ Section of Metabolic Diseases Beatrix Children's Hospital, University Medical Center Groningen, University of Groningen Groningen The Netherlands; ^2^ Department of Endocrinology University of Groningen, University Medical Center Groningen Groningen The Netherlands; ^3^ Laboratory of Pediatrics University of Groningen, University Medical Center Groningen Groningen The Netherlands

**Keywords:** carnitine, fatty acid oxidation, medium‐chain acyl‐CoA dehydrogenase deficiency, secondary carnitine deficiency

## Abstract

Our aim was to study the effect of secondary carnitine deficiency (SCD) and carnitine supplementation on important outcome measures for persons with medium‐chain Acyl–CoA dehydrogenase deficiency (MCADD). We performed a large retrospective observational study using all recorded visits of persons with MCADD in the University Medical Center Groningen, the Netherlands, between October 1994 and October 2019. Frequency and duration of acute unscheduled preventive hospital visits, exercise tolerance, fatigue, and muscle pain were considered important clinical outcomes and were studied in relation to (acyl)carnitine profile and carnitine supplementation status. The study encompassed 1228 visits of 93 persons with MCADD. >60% had SCD during follow‐up. This included only persons with severe MCADD. Carnitine supplementation and SCD were unrelated to the frequency and duration of the acute unscheduled preventive hospital visits (*P* > 0.05). The relative risk for fatigue, muscle ache, or exercise intolerance was equal between persons with and without SCD (RR 1.6, 95% CI 0.48–5.10, *P* = 0.4662). No episodes of metabolic crisis were recorded in non‐carnitine‐supplemented persons with MCADD and SCD. In some persons with MCADD, SCD resolved without carnitine supplementation. There is absence of real‐world evidence in favor of routine carnitine analysis and carnitine supplementation in the follow‐up of persons with MCADD.

## 1 INTRODUCTION

Medium‐chain Acyl–CoA dehydrogenase deficiency (MCADD) is one of the most common inborn errors of metabolism. The disorder has had a prevalence of 1:8000 in the Netherlands since the introduction of population newborn bloodspot screening (NBS).^1^, ^2^ MCADD impairs the mitochondrial β‐oxidation of medium‐chain fatty acids (C6–C12), which leads to a risk of developing hypoketotic hypoglycemia during periods of high metabolic demand and low carbohydrate supply. Biochemically, MCADD is associated with abnormal (acyl)carnitine (AC) plasma profiles. Increased octanoyl‐ (C8), decanoyl‐ (C10) carnitine concentrations, and their molar ratio are most characteristic and form the basis of NBS for the disorder.^3^ Based on the *ACADM* genotype, residual MCAD enzyme activity, and acylcarnitine profiles, “severe” and “mild” subtypes are distinguished.^1^, ^2^, ^4^ The basic principles of management for all persons with MCADD consist of the avoidance of fasting and an emergency regimen.^5^, ^6^, ^7^, ^8^

Carnitine (L‐3‐hydroxy‐4‐*N‐N‐N*‐trimethylaminobutyrate) facilitates the transport of activated long‐chain fatty acids from the cytoplasm to the mitochondrial matrix. In non‐vegetarians, approximately 75% of carnitine is provided by diet (animal products) and 25% from de novo biosynthesis from the amino acids' lysine and methionine. Only 0.5% of the carnitine circulates; plasma levels are very low (40–60 μmol/L) compared to tissue concentrations (2–3 mmol/kg in the muscle up to 800–1500 mmol/kg in the liver).^9^, ^10^ After ingestion or synthesis, carnitine is not further metabolized and filtered by the glomerulus. Approximately 95% of the free carnitine is reabsorbed in the proximal tubule, while ACs are excreted. The sodium‐dependent carnitine transporter OCTN2 is the most important carnitine transporter. OCTN2 controls the body carnitine pool and defines the renal excretion threshold for free carnitine, which normally lies around 50 μmol/L. In persons with MCADD, low plasma concentrations of free carnitine, or “secondary carnitine deficiency” (SCD), are observed.^1^ Accumulating ACs are thought to deplete the carnitine stores because of increased urinary excretion and an inhibitory effect on OCTN2, lowering the renal excretion threshold for free carnitine.^11^, ^12^

Carnitine supplementation is still controversial in persons with MCADD.^1^, ^13^ Moreover, the natural longitudinal course of plasma (acyl)carnitine concentration in persons with MCADD is unknown.^7^ In the absence of randomized controlled trials, observational studies evaluating the effectiveness of a treatment using disease‐specific outcomes can provide relatively high‐quality evidence.^14^, ^15^ We questioned whether SCD and/or carnitine supplementation is associated with the clinical outcome of persons with MCADD. Therefore, we conducted a retrospective observational cohort study in persons with MCADD to describe the natural longitudinal course of plasma carnitine concentrations and the effect of carnitine supplementation. The frequency and duration of acute unscheduled preventive hospital visits, exercise tolerance, fatigue, and muscle pain were considered important clinical outcomes.

## 2 METHODS

### 2.1 Ethics approval

The Medical Ethical Committee of the University Medical Center Groningen stated that the Medical Research Involving Human Subjects Act was not applicable and that official study review and approval by the Medical Ethical Committee were not required (METc 2019/119).

### 2.2 Study population

This was a monocenter, retrospective, observational cohort study of the plasma carnitine concentrations and clinical outcomes of all persons with MCADD with a recorded visit in the electronic health record system of the University Medical Center Groningen, Groningen, the Netherlands, between October 1994 and October 2019. Persons were excluded when (a) the diagnosis was established after death, (b) no plasma (acyl)carnitine profile was analyzed in our center, or (c) severe comorbidity likely interfered with outcome measures. Severe MCADD was defined by an *ACADM* genotype historically associated with clinical ascertainment, *or* residual MCAD activity <10%. Mild MCADD was defined by an *ACADM* genotype that has never been associated with clinical ascertainment *and* residual MCAD activity >10%, as previously published.^2^, ^4^, ^16^ All data were retrieved from the electronic health record system of the University Medical Center Groningen, Groningen, the Netherlands. Data of admissions in other centers were based on archived correspondence and/or discussion of interim admissions during regular out‐patient clinic visitse.

### 2.3 Treatment

Management of our MCADD population is based on the national clinical care pathway, of which the first version dated from 2012, and was recently updated.^18^ In brief, it consists of generalized age‐dependent advice on avoidance of fasting, and prevention of metabolic emergencies, as published elsewhere.^8^, ^17^ It is advised to assess the AC profile at least yearly and at indication (i.e., complaints of fatigue, exercise intolerance, or muscle ache). Carnitine supplementation is considered in the first 4 years of life if low‐plasma free‐carnitine concentrations are present, or during illness (Derks et al. 2020). Dosing standards are not explicitly defined.^1^, ^18^

### 2.4 Outcome measures

Concentrations of TC‐ (total), C0‐ (free), C2‐ (acetyl‐), C6‐ (hexanoyl‐), C8‐ (octanoyl‐), and C10‐ (decanoyl‐) carnitine, as well as the C8/C10 ratio, were obtained from dried blood spots or blood plasma samples by flow‐injection tandem mass spectrometry.^19^ When multiple (acyl)carnitine profiles were analyzed on the same day, average values were taken. Carnitine deficiency was defined by a free carnitine concentration equal to or lower than the 2.5th percentile found in the age and year cohort‐specific control population (Table S1). (Acyl)carnitine profiles were studied in relation to patient characteristics (age, gender, and disease severity); carnitine supplementation (supplemented and not supplemented/not explicitly recorded); visit type (outpatient clinic visit, acute unscheduled preventive hospital visit, or research visit); specific complaints (fatigue, muscle ache, and exercise intolerance); acute unscheduled preventive hospital visits (in total amount, days, and categorized as by zero, one, or two and more); and pregnancy.

### 2.5 Data analysis

Data were divided into age categories corresponding to important changes in growth and feeding patterns: neonates (0–6 days), “full lactation,” that is, receiving frequent feedings (6–8 bottles/day) and presenting with rapid growth (7–55 days), infants (56 days to 19 months), preschool (20 months to 4 years), school age (5–10 years), adolescence (11–17 years), and adulthood (18–80 years), based on previous studies on age‐related differences in AC concentrations.^20^, ^21^

All analyses were performed using R (Version 4.0.3, R Foundation for Statistical Computing, Vienna, Austria). Descriptive statistics were used for all continuous data including means, medians, ranges (min‐max), standard errors (SE), and 95% confidence intervals (95% CI). Comparisons between groups with normally distributed data were done using the Student's T‐test if variances were equal, and with Welch two‐sample t‐test, if variances were unequal. In the case of non‐normal distribution, groups were compared using Wilcoxon rank‐sum tests with continuity correction.

To study possible influences on the most disease‐specific AC for MCADD, we fitted linear mixed‐effect models (LMMs) with normalized total C, C0‐, C2‐, C6‐, C8‐, C10‐, and C8/C10 ratio using the “lme4” (Bates, Maechler & Bolker, 2015) and “lmerTest” *(*Kuznetsova, Brockhoff & Christensen, 2017*)* R packages. Linear mixed modeling was chosen as it can handle the non‐independent nature of our data (hence, it consists of repeated measures in the same persons) and the missing data (occurring due to the retrospective nature of our study). As fixed effects, we entered disease severity, gender, and the number of acute unscheduled preventive hospital visits (0, 1, or >1) in the period before the visit into the model, without interaction terms. As the random effects, we entered patient and age categories (as described above 2.5). Model assumptions were verified by visual inspection of residuals versus fitted values and residuals Q‐Q plots. To meet the assumption of normality, AC data were normalized by log transformation. *P*‐values were obtained by restricted maximum likelihood ratio tests of the full model with effect in question against the model without effect in question. *P*‐values < 0.05 were considered significant.

## 3 RESULTS

### 3.1 Cohort

**FIGURE 1:**
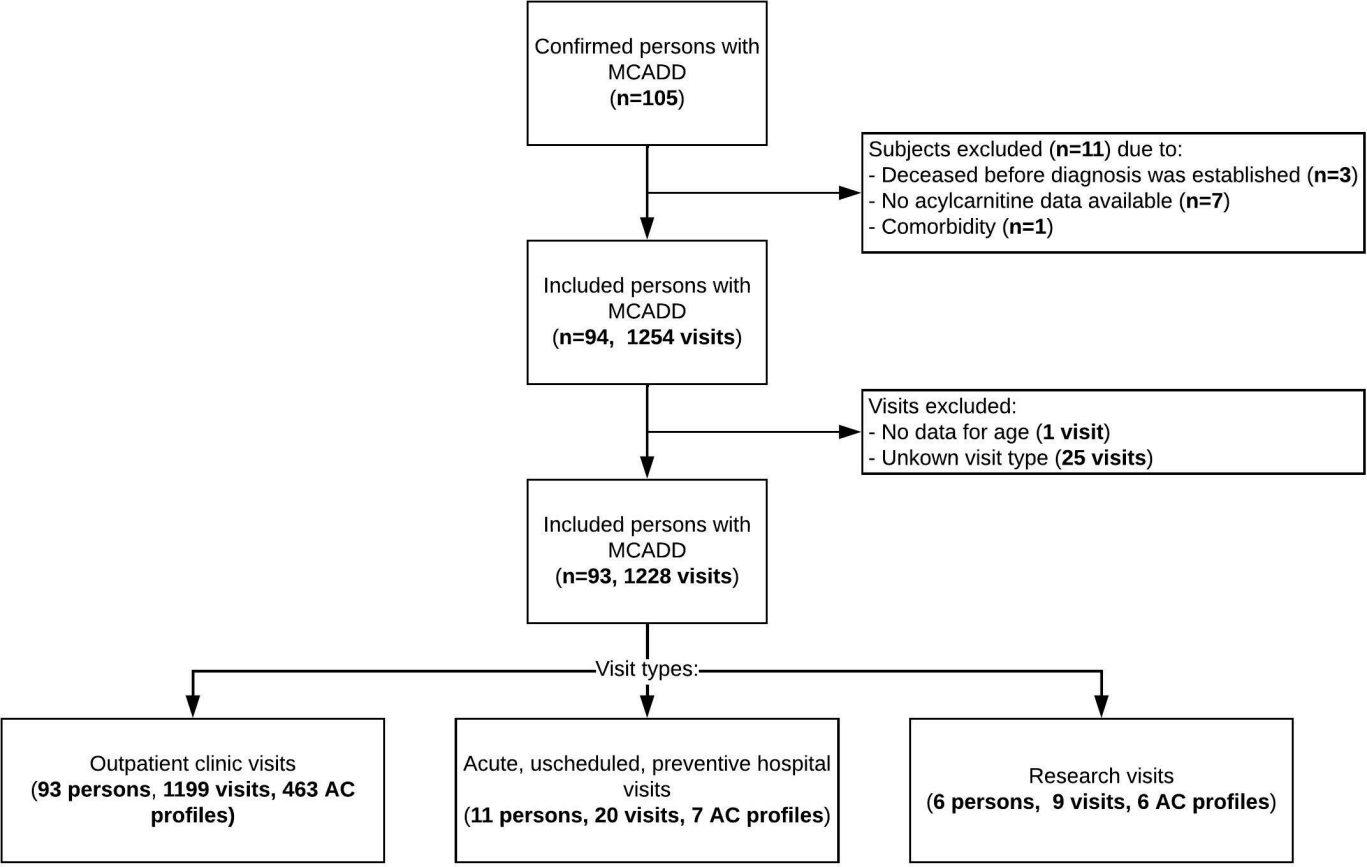
Flowchart of cohort selection and categorization of the final study cohort. “N” shows the number of unique persons with MCADD. “Unscheduled hospital admissions” represent acute unscheduled, preventive hospital admissions in our center specifically (in the context of the emergency regimen), to prevent metabolic derangement. “AC” shows the number of (Acyl)carnitine profiles established during the visits

The cohort encompassed 1228 visits of 93 persons with MCADD (representing 959 years of follow‐up) (Figure 1). This included 42 males and 51 females, of whom 86 persons were categorized as severe and seven as mild. The median follow‐up was 11 years (range: 0 days – 24 years). A vegetarian diet was explicitly recorded in only one patient, during one visit. Four persons were excluded from the study. Three persons had deceased before diagnosis was established (Table S2), whereas one patient had comorbidity of presynaptic congenital myasthenic syndrome‐7B (CMS7B, OMIM:#619461) (c.805G > T (p.[Glu269*])) *SYT2* (OMIM:*600104), which led to long‐term hospitalization, mechanical ventilation, and prolonged nasogastric feeding. The case is described in detail elsewhere.^22^

**FIGURE 2:**
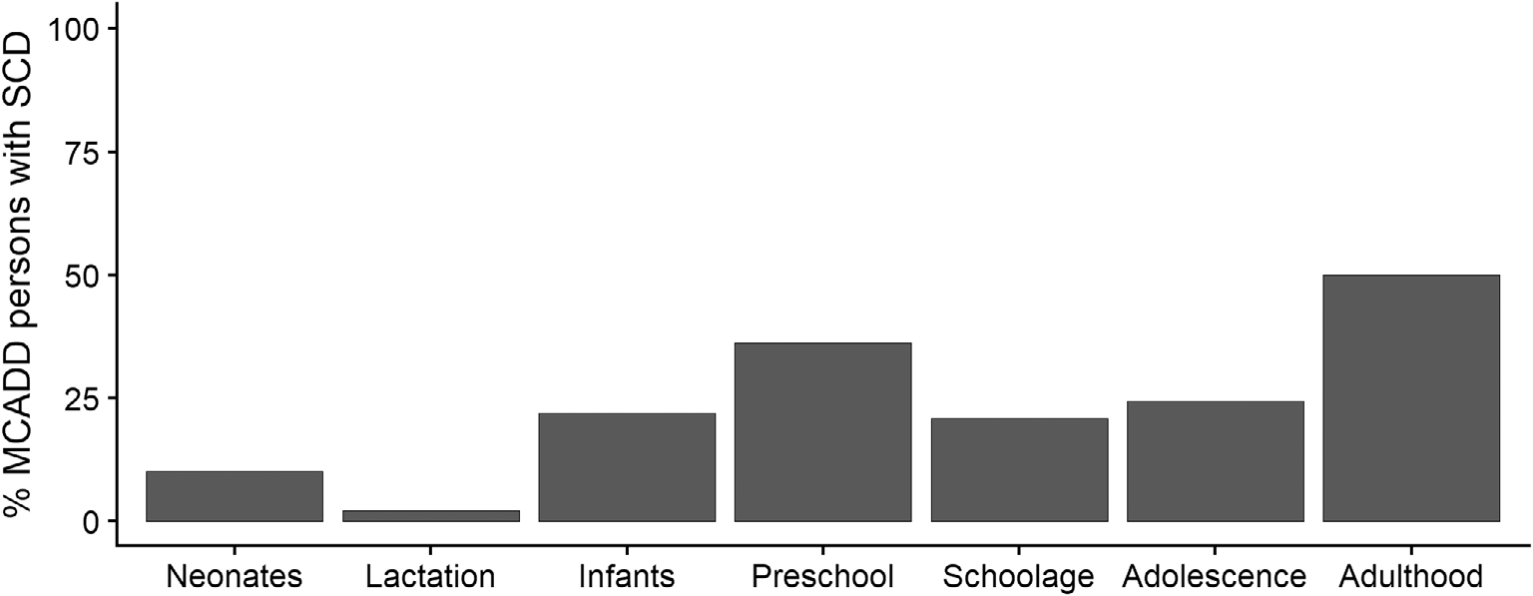
Percentage of persons with MCADD and secondary carnitine deficiency by age category. The figure depicts the percentage of persons with MCADD in which SCD was established at least once in the given age category by total persons included per age category

A total of 476 AC profiles were analyzed, equaling a mean of 1 carnitine profile per patient every 2 years of follow‐up. Most persons with MCADD (61%; 57/93) displayed SCD at some point during follow‐up. This included only persons with severe MCADD. SCD was found in 24% of the measured profiles (116/476). SCD was relatively most prevalent among preschoolers and adults (Figure 2). The three AC profiles analyzed during pregnancy showed (borderline) SCD C0 concentrations. Prevalence of SCD remained high in adults (46%), even when ignoring pregnant women. SCD was equally present in males and females (males *n* = 25/57, females *n* = 32/57).

Carnitine supplementation was recorded for 61% (57/93) of persons during 392 visits. On average, a carnitine dose of 35 mg/kg/day was given (range: 0.4–234 mg/kg/d). In 21% (24/116), SCD was observed during a visit despite carnitine supplementation (doses ranging from 8 to 38 mg/kg/d).

### 3.2 Acute unscheduled preventive hospital visits

**FIGURE 3:**
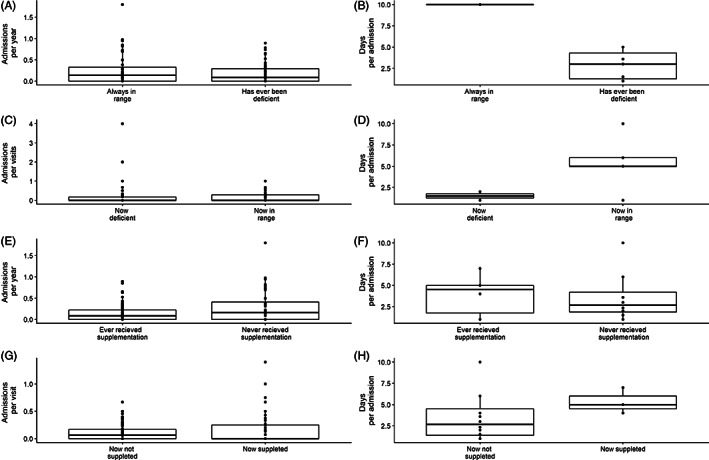
Effect of secondary carnitine deficiency and carnitine supplementation on frequency and duration of acute unscheduled preventive hospital visits. (A) The number of admittances per person, per year of follow‐up, and (B) the duration in days per admission between persons who have been carnitine deficient at some point during follow‐up (Has ever been deficient), compared to persons who were never carnitine deficient during follow‐up (Always in range) (*P*‐value = 0.26 and *P*‐value = 0.19 respectively, Wilcoxon rank‐sum test with continuity correction). (C) The number of admittances per person, per total visits their C0 concentrations was in range or deficient, and (D) duration in days per admission are comparable between persons with C0 concentrations within in range (Now in range) versus those with deficient C0 concentrations (Now deficient) (*P*‐value = 0.809, Wilcoxon rank‐sum test with continuity correction and *P*‐value = 0.3272, Welch two‐sample t‐test). (E) The number of acute unscheduled preventive hospital visits per person per year of follow‐up, as well as (F) duration in days per visit is equal between persons who have had carnitine supplementation (Ever received supplementation) at some point during follow‐up versus persons who have never received supplementation (Never received supplementation) during follow‐up (*P*‐value = 0.1327, Wilcoxon rank‐sum test and *P*‐value = 0.9165, Welch two‐sample t‐test). (G) The number of admittances per person per visit, as well as (H) the duration in days per visit are comparable between persons who received carnitine supplementation (Now supplemented) at that moment versus those who did not receive carnitine supplementation at that moment (Now not supplemented) (*P*‐value = 0.3786, Wilcoxon rank‐sum test with continuity correction and *P*‐value = 0.1467, Wilcoxon rank‐sum test with continuity correction). Box shows median, first, and third quartiles. Whiskers extent to smallest and highest values, until 1,5 × the interquartile range (IQR). All data points are also plotted individually (●)

In our cohort, 35 persons never required an acute unscheduled, preventive hospital visit. The other 57 persons with MCADD were admitted 170 times to prevent a metabolic crisis (median frequency 2, range 1–10). Gastroenteritis, fever, and vomiting were the main reasons for admittance. The median age at acute unscheduled preventive hospital visits was 2.0 years (range: 4 days to 27 years). Carnitine supplementation and SCD were not related to the frequency and duration of the acute unscheduled preventive hospital visits (*P* > 0.05) (Figure 3). We could not derive a clear association between SCD or carnitine supplementation and the acute unscheduled preventive hospital visits of the MCADD persons with the most severe clinical presentations (hypoglycemia or seizures), or the most frequent acute unscheduled preventive hospital visits (range 8–10 times).

**FIGURE 4:**
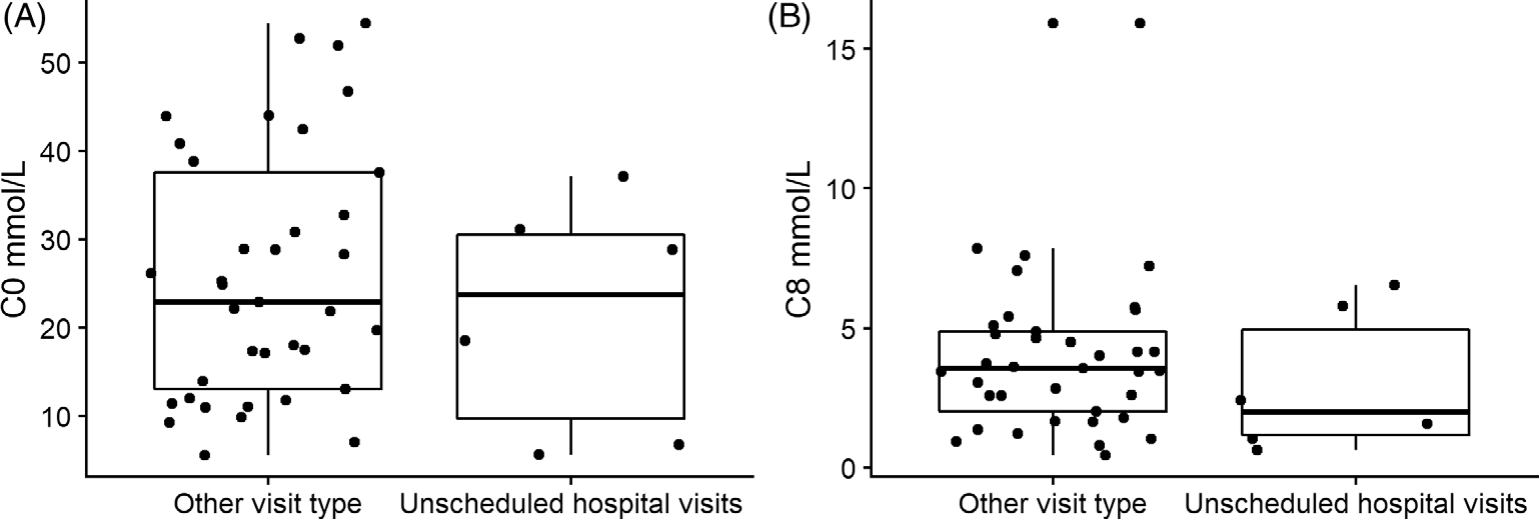
Acylcarnitine concentrations during out‐patient' clinic visits compared to acute unscheduled preventive hospital visits. Comparison of (A) C0 concentrations and (B) C8 concentrations during outpatient clinic visits and acute unscheduled preventive hospital visits. Boxplots include (acyl)carnitines values during visits in which carnitine was not supplemented. Box shows median, first, and third quartiles. Whiskers extent to smallest and highest values, until 1,5 × the interquartile range (IQR). All data points are also plotted individually (●)

Detailed clinical information on acute unscheduled preventive hospital visits was rarely available because hospitalization occurs mostly in local hospitals. Therefore, we were only able to study 7 AC profiles of five persons with acute unscheduled preventive hospital visits in our center. C0‐ and C8‐carnitine values measured during these acute unscheduled preventive hospital visits were similar compared to values during regular out‐patient clinic visits. Only values derived from persons not receiving carnitine supplementation were used in this analysis (*P*‐value = 0.549 and *P*‐value = 0.3714, respectively, Wilcoxon rank‐sum test with continuity correction) (Figure 4).

### 3.3 Fatigue, muscle ache, and exercise intolerance

Specific symptoms such as fatigue, muscle ache, and exercise intolerance were recorded in 38 visits of 18 persons, all with severe MCADD. The median age at which these symptoms occurred was 12 years (range: 2.5–43 years). In 11 of these 18 persons, during 23 of the 38 visits, carnitine was supplemented with doses ranging between 7 and 46 mg/kg/d. The relative risk (RR) for these specific complaints during a visit while receiving carnitine supplementation is 3.2 (95% CI 1.7–6.2, *P* < 0.001), compared to non‐supplemented visits. In three persons, carnitine supplementation was (re)started or the dose was increased specifically for these symptoms. All reported improvement of complaints within 3–4 months, however, acylcarnitine profiles were not systematically analyzed before, during, and after symptoms. In the other eight persons, the indication for carnitine supplementation was either previous low‐normal C0 concentrations, previous SCD, or not documented.

Acylcarnitine profiles were analyzed in 12 visits in which these specific MCADD symptoms were noted. In 33% (4/12) of these visits, SCD was found, with C0‐carnitine concentrations of 13.7, 5.4, 15.2, and 13.7 μmol/L (reference range: 16–55 μmol/L). The latter three profiles were analyzed while carnitine was supplemented in the respective doses of 7, 30, and 24 mg/kg/d. RR for specific complaints is equal for persons during visits where they have SCD compared to those having C0‐carnitine concentration in the range (RR 1.6, 95% CI 0.48–5.10, *P* = 0.4662). Conditional means of the C0 concentration are equal during life in persons with MCADD exhibiting and not exhibiting these symptoms at some point during follow‐up, irrespective of supplementation status (Figure S1).

### 3.4 Acylcarnitine profiles without carnitine supplementation

**FIGURE 5:**
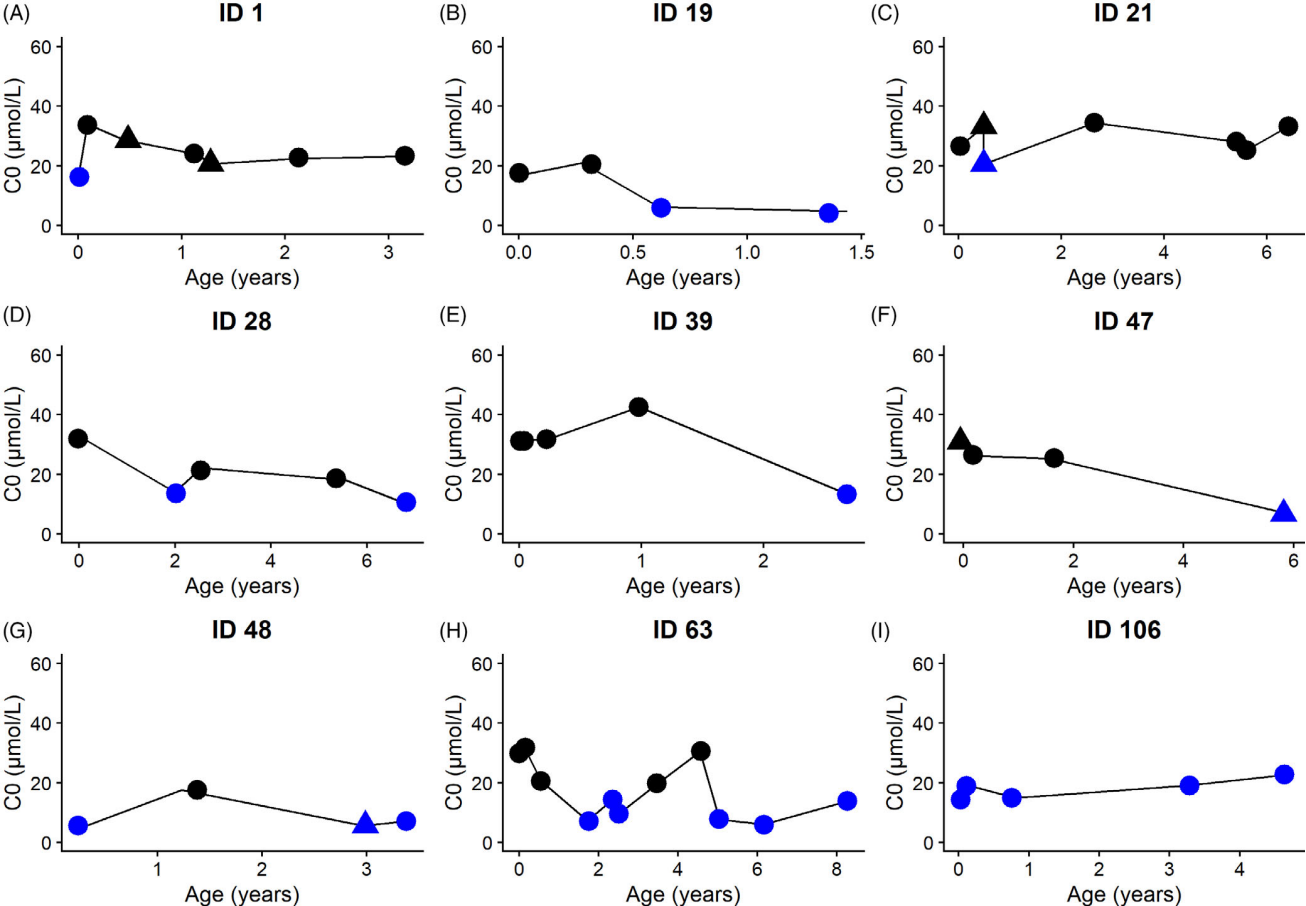
Course of C0 concentrations during follow‐up of persons with MCADD with SCD who never received carnitine supplementation. Black color indicates a C0 within the normal range, and blue indicates C0 deficiency. Dots (●) represent out‐patient clinic visits, squares (■) represent research visits, and triangles (▲) show acute unscheduled preventive hospital visits. ID numbers correspond to specific persons

A total of 9/36 persons with MCADD who never received carnitine supplementation throughout follow‐up, developed SCD. Sixty‐six percent (6/9) were found deficient during multiple visits. No episodes of acute severe metabolic crisis were recorded in these persons (such as hospitalization because of severe hypoglycemia, seizures, coma, or death). C0 concentrations fluctuated and sometimes normalized without interference (Figure 5).

The results of LMMs for disease‐specific AC can be seen in Table 1. This analysis is based on AC profiles of persons with MCADD in which carnitine never has been supplemented. R^2^ ranged from 0.235 for C10 to 0.933 in C8/C10.

**TABLE 1:** Results of generalized mixed models of acylcarnitine profiles

	TC				C0				C2				C6				C8				C10				C8/C10 Ratio			
*Predictors*	*Estimates*	*se*	*CI*	*T value*	*Estimates*	*se*	*CI*	*T value*	*Estimates*	*se*	*CI*	*T value*	*Estimates*	*se*	*CI*	*T value*	*Estimates*	*se*	*CI*	*T value*	*Estimates*	*se*	*CI*	*T value*	*Estimates*	*se*	*CI*	*T value*
(Intercept)	3.70	0.14	±0.28	26.28	3.44	0.17	±0.34	20.23	1.61	0.19	±0.38	8.07	−1.55	0.24	±0.48	−6.39	−0.43	0.30	±0.59	−1.43	−1.38	0.21	±0.41	−6.59	0.93	0.20	±0.41	4.61
Severity (Mild)	−0.32	0.13	±0.25	−2.56	−0.46	0.16	±0.31	−2.96	−0.45	0.16	±0.31	−2.87	0.71	0.22	±0.44	3.19	1.46	0.30	±0.60	4.86	−0.02	0.19	±0.38	−0.09	1.61	0.21	±0.42	7.67
Sex (Female)	−0.00	0.10	±0.20	−0.02	−0.04	0.12	±0.24	−0.33	−0.06	0.13	±0.25	−0.47	0.21	0.18	±0.35	1.17	0.16	0.24	±0.46	0.66	0.09	0.15	±0.30	0.59	−0.03	0.17	±0.33	−0.19
Single Admittance	−0.15	0.09	±0.19	−1.56	−0.08	0.11	±0.22	−0.73	−0.15	0.15	±0.31	−1.09	−0.13	0.17	±0.25	−0.74	−0.24	0.21	±0.41	−1.15	−0.19	0.18	±0.35	−1.04	−0.08	0.06	±0.12	−1.19
Multiple admittances (2 or more)	0.27	0.36	±0.71	0.75	0.51	0.42	±0.81	1.22	−0.35	0.59	±1.16	−0.59	0.24	0.67	±1.32	0.35	0.34	0.79	±1.57	0.43	0.36	0.71	±1.40	0.50	0.28	0.24	±0.47	1.18
**Random Effects**																												
σ^2^	0.11				0.15				0.30				0.39				0.54				0.45				0.05			
τ_00_	0.05 _Pat_nm_				0.08 _Pat_nm_				0.04 _Pat_nm_				0.14 _Pat_nm_				0.32 _Pat_nm_				0.06 _Pat_nm_				0.23 _Pat_nm_			
	0.04 _Age_category_				0.05 _Age_category_				0.09 _Age_category_				0.08 _Age_category_				0.04 _Age_category_				0.07 _Age_category_				0.00 _Age_category_			
ICC	0.43				0.45				0.29				0.37				0.40				0.23				0.83			
N	36 _Pat_nm_				36 _Pat_nm_				36 _Pat_nm_				36 _Pat_nm_				36 _Pat_nm_				36 _Pat_nm_				36 _Pat_nm_			
	7 _Age_category_				7 _Age_category_				7 _Age_category_				7 _Age_category_				7 _Age_category_				7 _Age_category_				7 _Age_category_			
Observations	133				135				129				130				135				134				134			
Marginal R^2^/Conditional R^2^	0.094 / 0.485				0.120 / 0.517				0.084 / 0.383				0.146 / 0.460				0.295 / 0.573				0.011 / 0.235				0.605 / 0.933			

*Note*: Generalized mixed‐effect models were made with AC profiles of never supplemented persons with MCADD (*n* = 35). CI is the 95% confidence interval of the estimate. Se is the standard error of the estimate. σ2 represents the mean random‐effect variance of the model. τ00 is the random intercept variance and indicates how much groups or persons differ from each other. ICC is the intraclass coefficient, which can be interpreted as the correlation among observations within the same cluster. Marginal R^2^ provides the variance explained only by fixed effects and conditional R^2^ provides the variance explained by the entire model (fixed effects as well as random effects).

**FIGURE 6:**
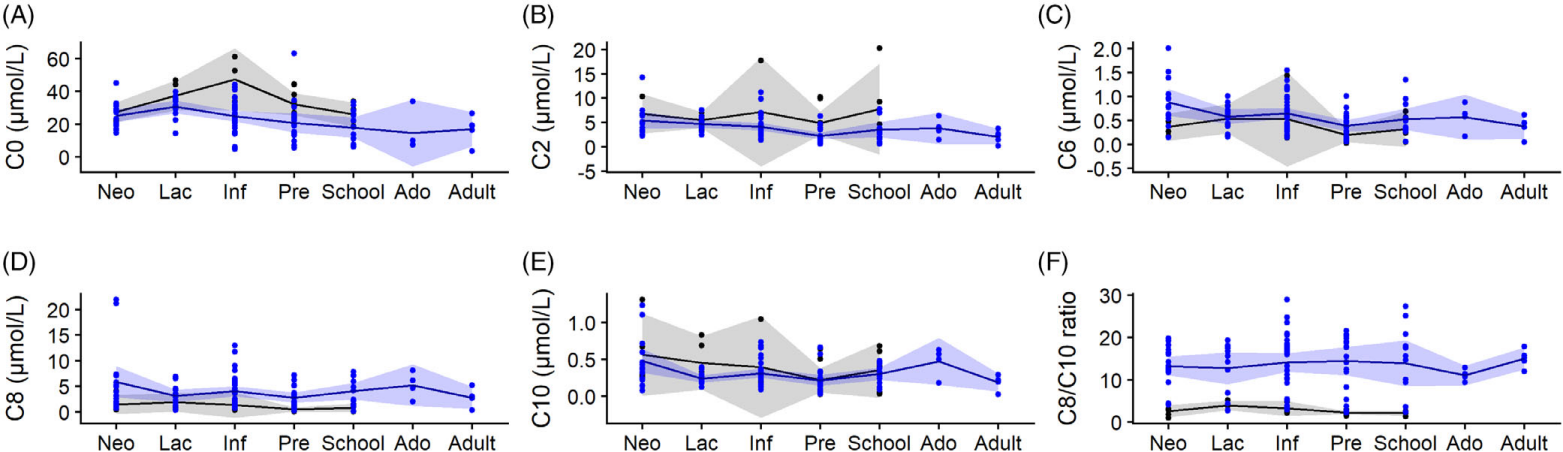
Acylcarnitine concentrations of persons with severe and mild MCADD by age category. Blue represents data of persons with severe MCADD (*n* = 29). Black represents data of persons with Mild MCADD (*n* = 7). Dots (●) represent individual acylcarnitine measurements. The lines represent mean concentration (by age category). The shading represents the 95% CI of the mean. The figure includes only acylcarnitine values established in persons who have never received carnitine supplementation during follow‐up

We found a significant effect of disease severity on AC profiles. TC was on average − 0.32 ± 0.25 log μmol/L (*P*‐value = 0.015) lower in persons with severe compared to mild MCADD. Also lower in severe persons with MCADD were concentrations of C0‐carnitine (−0.46 ± 0.16 log μmol/L, *P*‐value = 0.005) and C2‐carnitine (−0.45 ± 0.31 log μmol/L, *P*‐value = 0.008). Higher in severe persons were concentrations of C6‐carnitine (0.71 ± 0.44 log μmol/L, *P*‐value = 0.003), C8‐carnitine (1.46 ± 0.60 log μmol/L, *P*‐value < 0.001), and of the C8/10 ratio (1.61 ± 0.42 log, *P*‐value < 0.001). Sex and acute unscheduled preventive hospital visits did not affect the acylcarnitine concentrations. We found that TC, C0, and C6 concentrations were more similar per patient and per age category (e.g., the random effects both explained a significant part of the variation). Variation in C2 and C10 was only explained by similarity in concentration by age category. Variation in C8 and C8/C10 was only explained by similarities in concentration per patient. Figure 6 shows the AC profile time courses for these severe and mild persons with MCADD separately.

## 4 DISCUSSION

This study retrospectively evaluated SCD and carnitine supplementation in relation to relevant clinical outcomes over 25 years in a large and well‐documented MCADD cohort.^2^, ^4^, ^6^

SCD is considered a frequently observed comorbidity in persons with MCADD.^1^, ^23^ This study identified that more than 60% of the MCADD cohort had SCD during follow‐up. But, SCD only occurred in persons with severe MCADD, with the highest frequencies in preschoolers and adults.

The finding of SCD in preschoolers could be caused by the commonly occurring intercurrent illnesses in this age category. Low C0‐carnitine concentrations have been reported during a metabolic crisis.^24^ In the current study, C0‐carnitine concentrations were not found to be significantly decreased during acute unscheduled preventive hospital visits. This could be due to the early interventional, preventive character of our emergency regimen, in which frequent intake of a high carbohydrate solution has already started in the home setting.^8^ This is partly substantiated by the unaltered C8‐carnitine concentration, which is described to increase during fasting challenges.^16^, ^25^ However, we cannot exclude that increase in C8 concentrations was impeded by SCD in some cases, or that timing of the sampling during the admission may have influenced the profiles.^25^ Prognostic value of changes in C8 concentration remains to be established.

As acylcarnitine profiles were found to restore within 30 days after derangement and AC profiles were only established roughly once every 2 years in our cohort, the effect of acute unscheduled preventive hospital visits admittances on C0 and C8 concentrations may be underestimated when measured during out‐patient clinic visits.^24^ In earlier reports, decreasing C0‐carnitine concentrations in adult persons with MCADD had been attributed to the discontinuation of carnitine supplementation.^7^ We, however, found a decreasing trend in C0 concentration in severe persons with MCADD who never received carnitine supplementation (Figure 6). As many adult persons with MCADD are lost to follow‐up, a selection bias in AC analysis in adulthood is imaginable. The decreasing C0 concentration upon aging may well also be part of a physiological phenomenon. A negative correlation between C0 concentrations in muscle and age has been established in healthy humans, for which various etiological contributors are proposed, i.e., functional OCTN2 losses, drug interactions, and chronic diseases.^26^, ^27^ Sex hormonal changes have also been proposed to influence serum carnitine concentrations.^28^ In our study, we did not find sex‐related differences, which may partly be explained by the relatively young population of the included cohort. The C0 concentrations in the pregnant women with MCADD were in line with concentrations found in healthy women of similar gestational ages.^29^ Due to missing data, the dietary effects on (acyl) carnitine profiles could not be established. Normally, carnitine restricted diets and carnitine biosynthesis defects do not cause carnitine deficiency due to effective renal carnitine reabsorption.

In line with Wilson and co‐workers, this study did not observe cases of severe metabolic crisis after diagnosis associated with SCD and in the absence of carnitine supplementation.^5^ We found no correlation between the plasma carnitine concentrations or supplementation and the frequency, duration, and complications of acute unscheduled preventive hospital visits. However, information on the duration of admittance was only scarcely available. Previous studies reported that carnitine supplementation does not prevent hypoglycemia, vomiting, and lethargy upon fasting.^30^, ^31^

In our cohort, the relative risk of muscle ache, fatigue, and exercise intolerance during a visit was increased by carnitine supplementation. Only in three cases, it was recorded that carnitine was prescribed specifically for these symptoms. In these three cases, clinical improvement was reported, but the documentation was not structured or standardized. Future studies may incorporate these symptoms in a core outcome set and the efficacy of carnitine supplementation in symptomatic cases may be further studied in an N‐of‐1 approach.

Besides the absence of real‐world evidence in favor of carnitine supplementation in persons with MCADD, several considerations can be taken into account. First, the lack of a clear benefit of carnitine supplementation may be explained by the degree of carnitine deficiency. Based on the K_m_ of carnitine palmitoyltransferase‐1, *tissue* carnitine concentrations should be <10% of normal before they become a limiting factor for fatty acid oxidation.^10^ *Plasma* concentration of carnitine may not reflect the carnitine concentration in tissue. In studies performed in very long‐chain Acyl‐CoA dehydrogenase deficient (VLCADD) mice, increased liver carnitine concentrations were found in VLCADD mice versus healthy controls, despite lower plasma carnitine concentrations. The carnitine homeostasis in tissue may be maintained under metabolic stress through carnitine uptake from the plasma and stimulated carnitine biosynthesis.^32^, ^33^ Even in persons with primary systemic carnitine deficiency (or: OCTN2 deficiency, OMIM:#212140), who exhibit extremely low‐plasma (<5 μmol/L) carnitine concentrations, clinical manifestations range widely; from severe hypoglycemia early in life, development of cardiomyopathy later in life, to absence of symptoms.^34^, ^35^

The negative consequences of carnitine supplementation and carnitine profiling should also be considered. First, carnitine supplementation may aggravate the tissue accumulation of toxic AC in MCADD, as also seen in VLCADD mice.^36^ Second, carnitine supplementation may increase trimethylamine N‐oxide (a derivative of gut microbiota carnitine metabolism) which was recently discovered as a major risk factor for cardiovascular diseases.^37^ Lastly, repeated blood sampling is painful and stressful for children. Children are often regarded not to memorize the pain, but children already start self‐reporting pain from an age of 18 months. Distress from venipunctures can increase pain and decrease cooperation in subsequent medical procedures.^38^, ^39^ Strengthened by our finding that SCD can resolve without carnitine supplementation, we arrive at the same conclusion as Spiekerkoeter et al. and advise against routine carnitine analysis and supplementation in mild and asymptomatic persons with severe MCADD.^40^

LMMs of our (acyl)carnitine data showed great differences between persons with mild and severe MCADD. We advocate the separate biochemical analysis of severe and mild persons with MCADD, especially in longitudinal analysis. Since mild persons have only been identified since the expansion of the NBS, this may confound results.

We found C8 and C8/C10 to be relatively stable throughout life and within a patient, but they differed greatly between persons. Finding the origin of these individual differences between concentrations may also shed more light on the (patho)physiology of FAO(D). LMMs could prove of great value to the field of rare diseases in the future, as they can be adopted for the development of personalized medicine and as a data processing tool.^41^, ^42^

Some methodological remarks should be recognized. The retrospective character and large time period covered by this study have to be taken into account when interpreting the results. The content and detail of the reviewed clinical notes are biased due to differences in metabolic specialists and time‐related available knowledge. Furthermore, a lack of interoperability of electronic health records leads to missing data on (preventive) hospital admittances in centers other than our own. Although it is unlikely that major clinical events or severe complications would be missed, the patient cohort was closely followed, and shared care with the local hospitals is well organized. This does underline that already available information is left unused due to technical and organizational issues. In rare diseases, each patient file can be recognized as an individual case report containing invaluable information. Adoption of a general format to report disease‐specific core outcomes measures during out‐patient clinic visits, as well as better interconnectivity between electronic patient records will increase the possibilities for effectiveness trials in MCADD.^14^, ^15^

## 5 CONCLUSION

In conclusion, SCD is not associated with more frequent or prolonged acute unscheduled preventive hospital visits. It is recommended that (acyl)carnitine profiling and supplementation should only take place in persons with severe MCADD, *with* a clear clinical indication such as age‐inappropriate, unexplained, fasting intolerance, muscle weakness, or exercise intolerance. *If* carnitine supplementation is commenced, clinical and biochemical effects should be carefully monitored and documented by the treating physician.

## FUNDING INFORMATION

The MD/Ph.D. scholarship of Emmalie Jager (MD/Ph.D. 18/55) and Pilot Project of Merit Schaafsma (PP 19–65) are funded by the Junior Scientific Masterclass from the University of Groningen, University Medical Center Groningen.

## CONFLICT OF INTEREST

Emmalie Jager, Merit Schaafsma, Melanie van der Klauw, Rebecca Heiner‐Fokkema, and Terry Derks declare that they have no conflict of interest.

## INFORMED CONSENT

All procedures followed were in accordance with the ethical standards of the responsible committee on human experimentation (institutional and national) and with the Helsinki Declaration of 1975, as revised in 2000 (5). The Medical Ethical Committee of the University Medical Center Groningen stated that the Medical Research Involving Human Subjects Act was not applicable, official study approval by the Medical Ethical Committee was not required (METc 2019/119), and the study was approved for waived consent.

## ANIMAL RIGHTS

This article does not contain any studies with animal subjects performed by any of the authors.

## Supporting information


**Supplementary Figure S1** **: lifetime C0 concentrations of persons with MCADD who exhibited or not exhibited fatigue, muscle ache, or exercise intolerance during follow‐up.** A. C0 concentrations with and without carnitine supplementation combined. B. C0 concentrations while receiving carnitine supplementation C. C0 concentrations when not receiving carnitine supplementation. The figure only includes C0 concentrations of severe patients. Blue line indicates conditional mean of persons with MCADD who exhibited specific symptoms during follow‐up, and black line indicates persons with MCADD who did not exhibit specific symptoms during follow‐up. Gray area show 95% CI of the respective conditional mean (obtained by local polynomial regression).

**Supplementary Table S1**: Acylcarnitine reference values
**Supplementary Table S2**: Acylcarnitine profiles of deceased children with MCADD

## Data Availability

Data and material are available upon request from the corresponding author.
