# Supplementary figures and images for: Plasma carnitine concentrations in Medium‐chain acyl‐CoA dehydrogenase deficiency: lessons from an observational cohort study
Source: J Inherit Metab Dis. 2022 Jul 17;45(6):1118–29. doi: 10.1002/jimd.12537 (PMC9796739; doi:10.1002/jimd.12537)

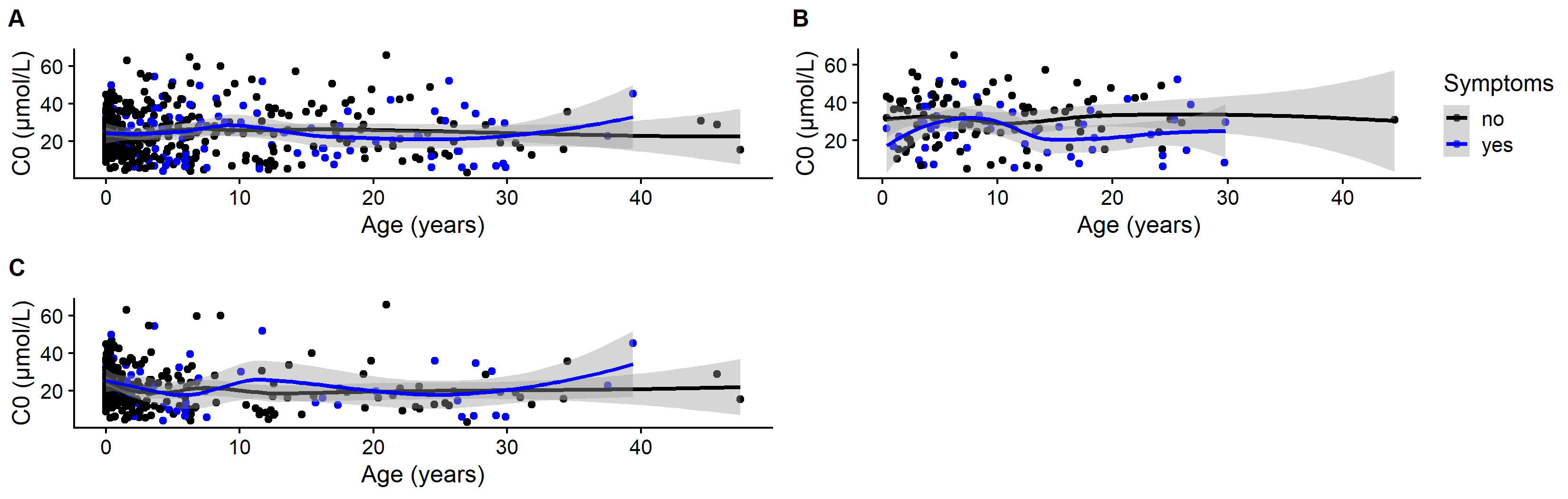

Supplement: Supplementary file 1 — Supplementary Figure S1 : lifetime C0 concentrations of persons with MCADD who exhibited or not exhibited fatigue, muscle ache, or exercise intolerance during follow‐up. A. C0 concentrations with and without carnitine supplementation combined. B. C0 concentrations while receiving carnitine supplementation C. C0 concentrations when not receiving carnitine supplementation. The figure only includes C0 concentrations of severe patients. Blue line indicates conditional mean of persons with MCADD who exhibited specific symptoms during follow‐up, and black line indicates persons with MCADD who did not exhibit specific symptoms during follow‐up. Gray area show 95% CI of the respective conditional mean (obtained by local polynomial regression). [file JIMD-45-1118-s002.tiff]
