# Supplementary material for: Plasma carnitine concentrations in Medium‐chain acyl‐CoA dehydrogenase deficiency: lessons from an observational cohort study
Source: J Inherit Metab Dis. 2022 Jul 17;45(6):1118–29. doi: 10.1002/jimd.12537 (PMC9796739; doi:10.1002/jimd.12537)
Supplement: Supplementary file 2 — Supplementary Table S1: Acylcarnitine reference values Supplementary Table S2: Acylcarnitine profiles of deceased children with MCADD [file JIMD-45-1118-s001.docx]

SUPPLEMENTARY TABLES

**Supplementary Table 1. Acylcarnitine reference values**

| **plasma µmol/l** | **1-7-2010** | **per 1-7-2010** | **per 1-1-2015** | | | **per 06-04-2016** | | |
| --- | --- | --- | --- | --- | --- | --- | --- | --- |
|  |  |  | 0-7 d | 7d-1mnd | > 1mnd | 0-7 d | 7d-1mnd | > 1mnd |
| total | 20-70 | 20-70 | 8-45 | 10-70 | 20-70 | 7-40 | 10-63 | 19-63 |
| C0 | 16-55 | 16-55 | 5-35 | 8-55 | 16-55 | 5-33 | 7-51 | 9-51 |
| C2 | 3.0-25 | 1.2-25.0 | - | - | 1.75-16.91 | 3.0-16.6 | 3.0-16.6 | 3.0-16.6 |
| C6 | 0.00-0.20 | 0.01-0.15 | - | - | 0.02-0.12 | 0.01-0.09 | 0.01-0.09 | 0.01-0.09 |
| C8 | 0.00-0.35 | 0.01-0.35 | - | - | 0.02-0.29 | 0.02-0.22 | 0.02-0.22 | 0.02-0.27 |
| C10 | 0.00-0.40 | 0.01-0.45 | - | - | 0.01-0.16 | 0.02-0.30 | 0.02-0.30 | 0.02-0.40 |
| C8/C2 | - | - | - | - | 0.00-1.00 | 0.0-0.1 | 0.0-0.1 | 0.0-0.1 |
| C8/C10 | - | - | - | - | 0.00-0.06 | 0.0-1.5 | 0.0-1.5 | 0.0-1.5 |
| C0/C total | 0.8 | 0.8 | 0.6-0.8 | 0.8 | 0.8 | 0.7-0.8 | 0.7-0.8 | 0.5-0.8 |

Mnd = month, d = days. <1mnd (n=10), > 1mnd (n=81)

**Supplementary Table 2: Acylcarnitine profiles of deceased children with MCADD**

| **ID** | **Genotype** |  | **Age**  (days) | **TC** | **C0** | **C2** | **C3** | **C4** | **C5** | **C6** | **C8** | **C10** | **C10:1** | **C12** | **C14** | **C14:1** | **C16** | **C18** | **C18:1** | **Remarks** |
| --- | --- | --- | --- | --- | --- | --- | --- | --- | --- | --- | --- | --- | --- | --- | --- | --- | --- | --- | --- | --- |
| B | c.985A>G | c.985A>G | 3 | 261 | 154 | 49,9 | 4.05 | 1.11 | 1.37 | 3,84 | 37,77 | 2,42 | 0,39 | 0,44 | 0,46 | 0,44 | 1,8 | 0,37 | 1,24 | Day of decease |
| K | - | - | 26 |  |  |  |  |  |  |  | 1,08 |  |  |  |  |  |  |  |  | Outcome pilot NBS screening |
|  |  |  | 56 |  |  |  |  |  |  | 0.95 | 4,46 | 0,44 | 0,46 |  |  |  |  |  |  | Follow-up diagnostics, decreased before results were available. |
| G | c.985A>G | c.985A>G | 3 |  |  |  |  |  |  |  |  |  |  |  |  |  |  |  |  | Diagnosis established in post-mortem tissue |

Acylcarnitines in µmol/L.
